# The Influence of Ferrule Design and Pulpal Extensions on the Accuracy of Fit and the Fracture Resistance of Zirconia-Reinforced Lithium Silicate Endocrowns

**DOI:** 10.3390/ma17061411

**Published:** 2024-03-20

**Authors:** Samah Saker, Ahmed Yaseen Alqutaibi, Mohammed Ahmed Alghauli, Danya Hashem, Sary Borzangy, Ahmed E. Farghal, Ahmad A. Alnazzawi, Sultan Ainoosah, Mohammed H. AbdElaziz

**Affiliations:** 1 Fixed Prosthodontics Department, Faculty of Dentistry, Mansoura University, Mansoura 35516, Egypt; samah_saker@hotmail.com; 2 Substitutive Dental Sciences Department, College of Dentistry, Taibah University, Al Madinah 41311, Saudi Arabia; sbarzanji@taibahu.edu.sa (S.B.); afrgal@taibahu.edu.sa (A.E.F.); anazawi@taibahu.edu.sa (A.A.A.); sainoosah@taibahu.edu.sa (S.A.); mhosseiny@taibahu.edu.sa (M.H.A.); 3 Prosthodontics Department, College of Dentistry, Ibb University, Ibb 70270, Yemen; malghawli100@gmail.com; 4 Restorative Dental Sciences Department, College of Dentistry, Taibah University, Al Madinah 41311, Saudi Arabia; dhashem@taibahu.edu.sa; 5 Fixed Prosthodontics Department, Faculty of Dental Medicine, Al-Azhar University, Cairo 11884, Egypt

**Keywords:** endocrowns, zirconia-reinforced lithium silicate, fracture resistance, accuracy, marginal and internal fit

## Abstract

The study aimed to assess the marginal, axial, and internal adaptation, as well as the fracture resistance of zirconia-reinforced lithium silicate (ZLS) endocrowns with varying pulpal inlay extensions and marginal geometry. Sixty extracted maxillary first molar teeth were divided into six groups (n = 10) according to pulpal inlay extension and marginal configuration. The first three groups (J2, J3, and J4) utilized prepared teeth for endocrowns without ferrule design and 2 mm, 3 mm, and 4 mm pulpal extensions, respectively. The second three groups (F2, F3, and F4) utilized prepared teeth with 1 mm shoulder margins and 2 mm, 3 mm, and 4 mm pulpal extensions. The endocrowns were fabricated from ZLS blocks using CAD/CAM milling technology. After cementation, the specimens underwent thermal aging for 5000 cycles and were evaluated for marginal adaptation. Using a universal testing machine, the fracture resistance was tested under quasistatic loading (1 mm/min). Two-way ANOVA and the Tukey’s post hoc test were employed for data analysis (*p* ≤ 0.05). The results of this study revealed that endocrowns without ferrule exhibited superior fracture strength than a 1 mm ferrule design *p* < 0.05, irrespective of the inlay depth. All designs with and without ferrule and all inlay depths showed clinically acceptable marginal and internal fit. The conventional endocrown design without ferrule and 2 mm inlay depth showed the lowest surface gap. The pulpal surface showed the highest discrepancy among all groups compared to the other surfaces. Endocrowns without ferrule are more conservative and have higher fracture strength than 1 mm ferrule designs; extending the inlay depth showed a significant increase in fracture resistance of the 1 mm ferrule design, but not for the conventional design without ferrule and 2 mm inlay depth. All groups exhibited a high auspicious fracture strength value for molar endocrown restorations.

## 1. Introduction

The severity of tooth structure loss of endodontically treated teeth (ETT) can significantly impact the treatment’s prognosis; the second most common cause of ETT extraction is vertical root fracture, primarily associated with post-placement [1,2]. The endodontic access cavity preparation removes tooth structure, weakens the cusps, and reduces their strength [3]. The clinical considerations of successful ETT are a combination of periodontal health and endodontic restoration perfection [4,5]. Although there is no evidence of reduced sensory function and proprioception of ETT, the non-vital teeth may receive a higher occlusal load than the vital teeth, as reported in a clinical study [6], so ETT possesses a higher probability of damage. Hence, enhancing ETT strength after the coronal seal is essential to guarantee clinical long-term survival [7].

Various procedures can be used to restore ETT, including direct composite restorations, indirect composite restorations, and metal and ceramic restorations. Many clinicians prefer indirect, full-coverage methods [8]. ETT with an extensive loss of hard tissue coronally might require a post-and-core crown, and that might be the only option; however, the presence of enough tooth structures and a minimum of two remaining axial walls would omit the need for root canal posts [9,10]. Recent advancements in dental adhesive materials and restoration fabrication technologies such as CAD/CAM, along with the development of contemporary ceramic materials, have led to the adoption of new and more advanced restorative approaches in dentistry. One of these evolutionary approaches is endocrown restoration, a conservative treatment approach that mitigates the failure risk by avoiding intracanal post-placement [11]. The endocrown is a monobloc all-ceramic restoration comprising a circumferential flat margin and an inlay cavity in the middle fitting the dental pulp chamber. It utilizes adhesive bonding and the available surface area in the pulp chamber to ensure the stability and retention of the restoration. This approach adheres to the concept of minimal invasive preparations [12]. The idea was first introduced in 1995 by Pissi and was called the Mono-block technique [13]. The idea was then modified to suit the posterior teeth by Bindl and Mörmann [14], pioneers of the endocrown as a terminology and concept for molar teeth. The design was further developed in a successive study usinga uniform and wide flat occlusal margin, 1–1.2 mm shoulder extension, and a 3 mm inlay depth, for less than 3 mm stump height, reduced preparation parameters were performed [15]. Endocrown restoration is an easy-to-perform treatment that conserves tooth structure, reduces treatment sessions and time, and increases patient satisfaction [16].

The original and recommended preparation design includes a 90° butt wide occlusal flat margin, which utilize the remaining tooth structure for restoration support and adhesion, a 6° internal axial walls taper with a flat pulpal floor, and supragingival enamel margins [15]. Including the ferrule feature in the preparation has enhanced the resistance to ceramic endocrown fracture [17,18]. Several ceramic materials have been utilized for endocrown restorations, oxide ceramics possess high strength to failure [17,19], meanwhile higher non-repairable failure rates [20], glass ceramics are the originally used endocrown restorations [13,14,15], and preferred regarding esthetic considerations, above all lithium disilicate, it has the highest flexural strength among glass–ceramics and offers the desired esthetic values [21]. Tetragonal zirconia fillers were incorporated into the microstructure to enhance the material flexural strength, resulting in zirconia-reinforced lithium silicate ceramics (ZLSs) [22]. However, another laboratory study reported higher fracture resistance for lithium disilicate endocrowns than ZLSs and resin nano-ceramic endocrowns under lateral and axial loading [23]. ZLS endocrowns reported higher fracture resistance than lithium disilicate endocrowns when loaded at 45 degrees [24]. Meanwhile, polymer-infiltrated ceramic, lithium disilicate, and ZLS mandibular molar endocrowns possess fracture resistance that exceeds the physiological limit of a normal person [25]. Moreover, polyetheretherketon endocrowns reported higher fracture resistance than lithium disilicate and ZLS [26].

Besides the material used for endocrown fabrications, there are several factors governing the fracture resistance of dental restorations, such as the tooth anatomy and type [27], the amount of remaining tooth structure [28], the accuracy and adaptation of the restorations [29], and the restoration design and extensions [17,30]. Extending the pulpal inlay depth to 5 mm in the pulp chamber seems to affect the scanning accuracy of the digital workflow, increasing the pulpal extensions of endocrowns need to be accompanied by most modern scanning devices such as Primescan for better accuracy results [31]. The endocrowns’ accuracy and adaptation might depend on the tooth type and form; a laboratory study reported that mandibular endocrowns showed better adaptation than maxillary endocrowns [32]. In terms of endocrown preparation designs, there are several laboratory studies executed to evaluate the effect of endocrown pulpal extensions on fracture resistance [33,34]; others extend the preparation on the axial surface utilizing ferrule preparation [17,18,35]. The presence of the ferrule design can improve fracture resistance by acting as a reciprocal support against laterally exerted stresses [17,18]. Moreover, increasing the pulpal extension enhances the fracture resistance of endocrowns based on recent evidence-based conclusions [29]. All these design variations have been studied independently on mandibular endocrowns. Therefore, the present study aimed to compare the effects of varying lengths of pulp inlays (2 mm, 3 mm, and 4 mm) and the inclusion of ferrules in the design. The null hypotheses for this study are as follows: (1) there will be no statistically significant difference in fracture resistance among endocrowns with different depths of pulp inlays. (2) The presence or absence of a ferrule will not affect the fracture strength of the endocrowns. (3) Varying depths of pulp inlays and the presence or absence of a ferrule will not impact the marginal, axial, and pulpal discrepancies of the endocrowns. (4) All designs, regardless of the presence of a ferrule and different pulp inlay extensions, will exhibit the same modes of failure.

## 2. Materials and Methods

### 2.1. Specimens Preparation

#### Teeth Selection, Preparation, and Grouping

The research protocol received approval from the Faculty of Dentistry ethics committee at Taibah University, with the assigned reference number 210323/TUCDREC for the year 2023. Freshly extracted, intact, and caries-free human maxillary molars were collected for the study. The teeth were inspected for preexisting visible chippings or cracks. The teeth were cleaned in an ultrasonic device and then transferred to a distilled water container at 4–5 °C until further use. The teeth with a similar dimension range were selected for the experiment (n = 60); the specimens’ mean dimensions were 10.47 ± 0.5 mm buccolingually and 11.13 ± 0.5 mesiodistally. 

The molar teeth were reduced horizontally to 3 mm above the most occlusal point of the cementoenamel junction (CEJ) using a diamond disc and a milling machine (BEGO. PARASKOP M.100-120, Bremen, Germany). Following the pulp morphology, the pulp chamber roof was removed using a round carbide high-speed bur. All teeth received endodontic treatment performed by the same operator (M.H) utilizing the protaper system (Dentsply-Maillefer; Ballaigues, Switzerland) and a standardized sequence with a 2.5% sodium hypochlorite irrigation solution. The teeth were held in wet gauze during the preparation and kept in a saline solution in between steps to prevent dehydration.

**Figure 1:**
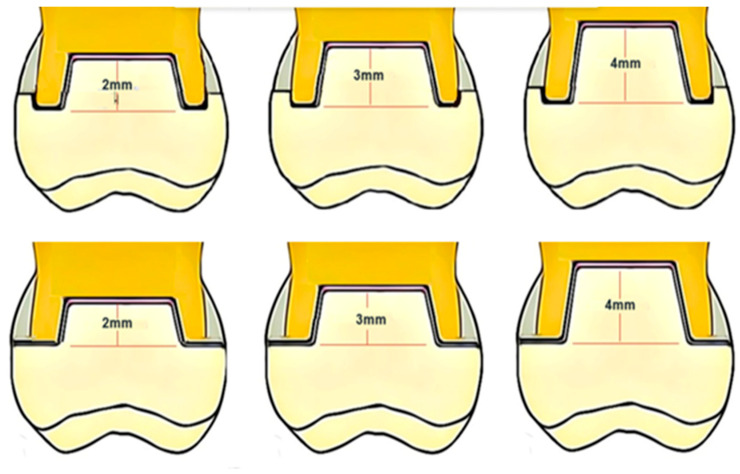
The preparation designs inlay depth differences and the presence of ferrule, upper raw 1 mm circumferential ferrule preparation with a shoulder finish line, lower raw with no ferrule preparation.

**Figure 2:**
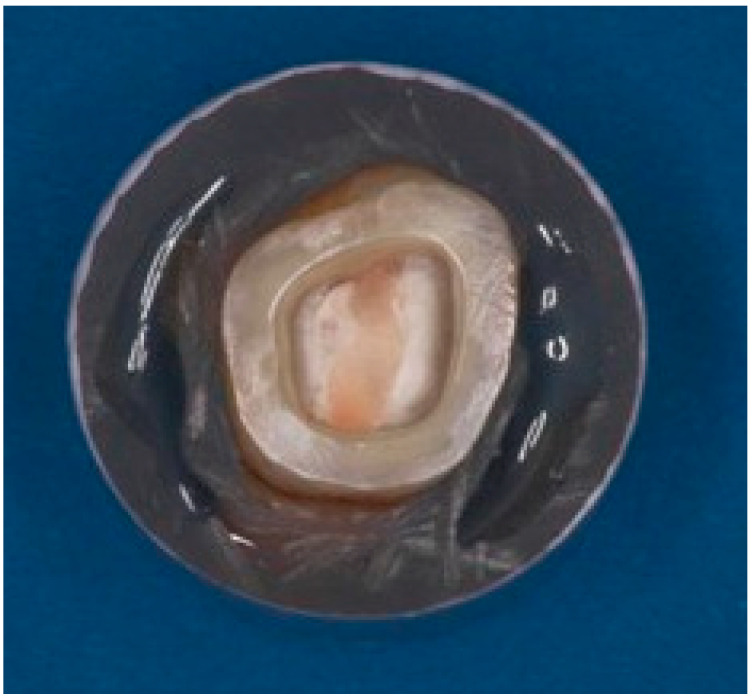
Endocrown preparation.

All excess sealer and debris were removed from the access cavity. The pulp chamber was cleaned using ethylene alcohol. All teeth were mounted in an auto-polymerizing acrylic resin (Ivocron; Ivoclar Vivadent AG, Schaan, Liechtenstein) in a standardized position parallel to their long axis and 3 mm apical to the CEJ. The pulp chamber cavity varied significantly from one tooth to another. The pulpal inlay was prepared with 8–10° divergence of the vertical walls. The discrepancies in the pulp chamber and the pulpal floor were restored using a two-step, self-etch adhesive (Clearfil SE; Kuraray America, Houston, TX, USA) and a dual-cure core material (Gradia Core; GC America, Alsip, IL, USA). The final preparation depth for the endocrown pulpal inlays was standardized at 2, 3, and 4 mm for the three major groups (n = 20), with a parallel pulpal floor to the endocrown occlusal table (Figure 1 and Figure 2).

A silicone stopper was used to standardize the depth of preparation, and the drill was equipped with a silicone stopper to measure the depth of the cavity. All-access cavities were modified to the same width (4 ± 0.2 mm buccolingually and 6 ± 0.2 mm mesiodistally). The teeth were further divided into two subgroups (n = 10) based on the type of external coronal preparation. Group N received no ferrule preparation, and Group F1 had 1 mm circumferential ferrule preparation with a shoulder finish line (Figure 1). All the preparation steps were completed with a parallelometer to ensure standardized preparation for all specimens.

The prepared teeth were scanned using a TRIOS 3 intraoral scanner (3Shape, Copenhagen, Denmark). The endocrowns were then designed with a 60-μm cement space using computer-aided design (CAD) software (3Shape CAD Design software, version 1.7.1.4, 3Shape) and were subsequently milled from ZLS blocks (VITA Suprinity, VITA Zahnfabrik, Bad Säckingen, Germany) using a CAD/CAM milling machine (Ceramill; Amann Girrbach AG, Koblach, Austria), following the manufacturer’s recommendations. To ensure consistency in the testing design, all restorations were designed with identical occlusal table anatomy and height, thereby minimizing the incorporation of different lever action vectors. A comprehensive list of the materials utilized in this study is provided in Table 1.

### 2.2. Marginal and Internal Adaptation Assessment

The marginal and internal adaptation of the restorations were assessed using the silicon replica technique. Each endocrown was loaded with a light-body impression material (President light body green; Colten, Konstanz, Germany) and seated for 5 min under a constant axial force of 50 N. After polymerizing the light-body material, the restoration was removed gently, and a heavy-body silicone (President’s heavy body) was injected into the tooth to stabilize the thin silicone film. Once polymerized, each replica was cut into four sections using a sharp surgical blade in a mesiodistal and buccolingual direction (no. 11; Feather Safety Razor Co., Ltd., Osaka, Japan). A 2-mm thick parallel wall slice was then sectioned from each piece to facilitate evaluation under a digital trinocular stereomicroscope (AmScope 3.5; Irvine, CA, USA) at ×50 magnification. Each slice was divided into four areas of interest for better comparison: axial, cervical, marginal, and pulpal floor.

Eight measurements were obtained on each slice: one marginal gap measurement, M1; two cervical area measurements (C1 in the center and C2 at the cervical–axial angle); three axial measurements (A1, A2, and A3); and two pulpal measurements (P1 on the axio-pulpal angle and P2 at the center of the pulpal area). The M1 measurement represented the marginal gap, while the internal adaptation of the restoration was expressed by the average of C1, C2, A1, A2, A3, P1, and P2. A total of 1920 measurements were taken for the 6 groups (8 measurements × 4 sections × 10 endocrowns × 6 groups).

### 2.3. Fracture Resistance Test

The internal surfaces of the endocrowns were etched with 5% hydrofluoric acid (IPS Ceramic Etching Gel; Ivoclar Vivadent) for 20 s, rinsed with water for 15 s, and dried with oil-free compressed air. A thin coat universal primer (Monobond Plus; Ivoclar Vivadent) was then applied to the etched intaglio surface using a micro brush for two 60-s intervals, with excess agent dispersed by compressed air. The prepared teeth were cleaned with fluoride-free pumice paste for 15 s and rinsed with water for 15 s. The endocrowns were then cemented with self-adhesive resin cement (Rely X Unicem, 3 M ESPE, Seefeld, Germany) and held under a constant axial load of 4.9 N exerted by a specially designed device for 5 min. After removing the excess cement, each side was light-cured for 20 s (Woodpecker iLED, Guilin, Guangxi, China, 2400 mW/cm^2^). The cemented endocrowns were stored in distilled water in a 37 °C incubator for 24 h. The specimens were then subjected to thermocycling at 5 °C to 55 °C and 15-s dwell time for 5000 cycles before undergoing the quasistatic load test.

**Figure 3:**
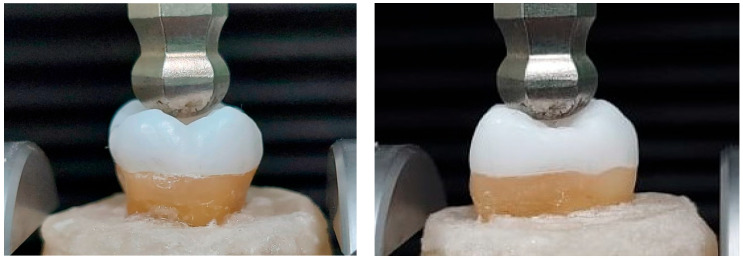
The quasistatic loading.

All specimens were loaded vertically along their long axis in a universal testing machine (Model 3345; Instron Industrial Products, Norwood, MA, USA) with a load cell of 5 KN at a speed of 1 mm/min until fracture, indicated by sudden drop of resistance, Figure 3. Each fractured specimen was visually inspected at 20× magnification (Hirox KH-7700, Hirox, Torrance, CA, USA) to determine the failure mode, which was classified as cohesive within the ceramic material, the adhesive between the ceramic and the tooth structure, or fracture of the tooth material. The modes were further categorized into favorable (repairable) or unfavorable (not repairable), based on agreement between two examiners. If the failure occurred above the cementoenamel junction (CEJ) and the cause of failure was only debonding and/or cohesive fracture of the restoration, or within the endocrown, it was considered a favorable fracture. Meanwhile, the tooth fracture below CEJ, including vertical root fracture, was considered unfavorable.

### 2.4. Statistical Analysis

Shapiro–Wilk’s and Levene’s tests were employed to test the assumption of normal distribution of the adaptation and resistance to fracture data. Given a non-significant result from the Levene test, indicating equal variances, a two-way analysis of variance (ANOVA) was conducted to evaluate the overall statistical significance of differences. A pair-wise statistical comparison was carried out using Tukey’s post-hoc test. Moreover, a Chi-squared test was conducted to assess the fracture modes. The statistically significant level was set at *p*-value ≤0.05.

## 3. Results

**Figure 4:**
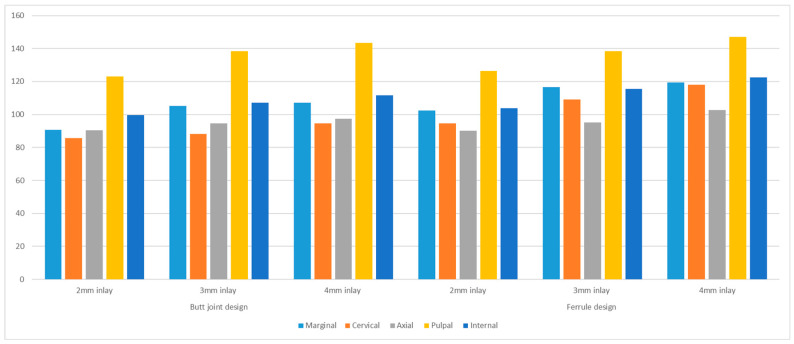
The bar chart shows the mean and standard deviation of the marginal, cervical, axial, pulpal, and internal gaps of endocrowns with different designs; the values are in µm.

All the data had a normal distribution according to the Shapiro–Wilk’s test *p* > 0.05. The mean values and standard deviations of marginal adaptation values for the tested groups are presented in Table 2 and Figure 4. The endocrowns with ferrule showed a statistically significantly higher gap at marginal, cervical, and internal surfaces: *p* = 0.002, <0.001, and <0.001, respectively. However, it was not statistically significant at the axial and pulpal surfaces, *p* = 0.323 and 0. 341, respectively. The largest gap was observed at the pulpal floor with a depth of 4 mm and a 1 mm ferrule marginal design *p* < 0.001, followed by the 3 mm inlay depth of both endocrowns with and without ferrule *p* < 0.001. The group with a 2 mm inlay depth recorded the highest adaptation. 

Regarding fracture resistance, endocrowns without ferrule groups showed higher resistance to failure than the ferrule design *p* < 0.001, irrespective of the pulpal inlay depth. 

The pulpal inlay depth 2 mm, 3 mm, and 4 mm showed a non-statistically significant effect on the resistance to fracture *p* = 0.265, 0.926, and 0.307, respectively. Table 3 presents the mean and standard deviations of resistance to fracture (N) for different marginal designs and pulp chamber depths (mm) of the ZLS endocrowns. Regarding the effect of the pulpal extension of the endocrowns, the results showed that restorations with a 3 mm pulpal extension exhibited higher mean fracture resistance values than 4 mm inlay depth, followed by endocrowns with a 2 mm pulpal extension. However, this difference was not statistically significant among the butt joint endocrowns without ferrule groups (*p* > 0.05). However, endocrowns with 1 mm ferrule designs showed lower fracture resistance for 4 mm deep inlay than 3 and 2 mm pulpal inlays, and all ferrule groups exhibited statistically significantly lower fracture resistance to the endocrowns without ferrule *p* < 0.001.

**Figure 5:**
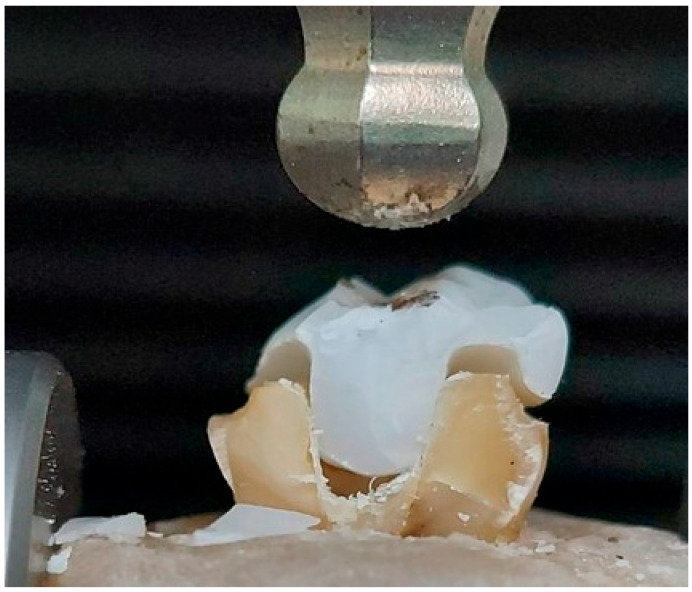
Unfavorable fractured specimen.

The Chi-squared test revealed no significant difference between the modes of failure of the tested groups. Unfavorable fracture was the most common mode of failure among all the tested groups, Figure 5. The specimens in the group with a chamber extension depth of 3 and 4 mm demonstrated almost universal catastrophic tooth fracture, while 70% of the specimens with a chamber depth extension of 2 mm showed unfavorable fracture.

## 4. Discussion

The rehabilitation of severely damaged ETT poses a significant challenge in dentistry. Recent advancements in restorative materials, adhesive protocols, and computer-aided design/computer-aided manufacturing (CAD/CAM) technology introduced endocrowns as a reliable and promising option for restoring ETT [15]. Numerous studies have emphasized the importance of effectively restoring ETT. The coronal hermitical seal with a successful coronal restoration meant not only to restore function and esthetics but to prevent the ingress of microorganisms to the obturated root canals [36,37,38]. Endocrown restorations bonded to coronal structure offer a viable, conservative, and time-efficient clinical option for sealing and restoring ETT [16].

In literature, most of the main bulk of data were on mandibular molar endocrowns or premolars [39]; in recent years, there have been more studies executed on maxillary molar teeth [32,40,41,42,43], all these records focused on the adaptation, marginal and internal discrepancies, as well as the retention and pullout tests. To the best of our knowledge, nearly all the existing records on fracture resistance and mechanical behavior of endocrowns have been conducted on mandibular molars. Only two studies were carried out on maxillary molars, and those studies used the conventional design without pulpal extensions or a ferrule design [44,45]. Due to anatomical variations in form, size of the pulp chamber, and location on the tooth, selecting maxillary molar teeth for this study appears highly beneficial. This will provide new data on endocrowns and establish a reliable reference for readers and clinicians involved in decision-making. This study investigated how pulp chamber extension depth and marginal design influence the accuracy of fit and resistance to fracture of mandibular molar endocrowns made from ZLS (VITA Suprinity) ceramics. The findings provided evidence to reject the null hypotheses, indicating that both variables, pulp chamber extension depths, and marginal design, significantly impact the accuracy of fit and resistance to fracture.

The findings showed that the resistance to fracture of ZLS endocrowns directly correlates to pulpal extension depth. Increasing depth shall increase the adhesion surface area enhance the distribution of stresses upon loading, and increasing the resistance to fracture. These findings were supported by previous studies by Dartora et al. 2018 [34] showing improved mechanical behavior with an increased pulpal extension of the endocrowns; the fracture resistance values were 2008.61 N, 1795.41 N, and 1268.12 N for 5 mm, 3 mm, and 1 mm inlay depth respectively. The fracture resistance of the current non-ferrule designs encountered 1371.1 N, 1409.6 N, and 1396.5 N for 2 mm, 3 mm, and 4 mm pulpal extensions, respectively.

On the other hand, Kuijper et al. 2020 [46] compared no extension overlay restoration to 2 mm and 4 mm pulpal extension endocrowns; the authors stated that there were no statistically significant differences between the fracture resistance of the three groups. However, the results of 2 mm and 4 mm groups were comparable and obviously higher than the overlay group, 812 ± 235 for the 0 extension, 1071 ± 408 N for 2 mm, and 1036 ± 278 N for the 4 mm extension [46]. The study tested the specimens after excessive fatigue loading, and the direction of the applied load was exerted at a 45° angle, simulating the parafunctional dynamic occlusion, unlike the current study, which tested the specimens without aging and directed the load in a vertical pattern simulating the centric occlusal contact, reflected upon the differences in the fracture resistance values of the two studies.

Moreover, a study by Hayes et al. 2017 [47] found that mandibular molars restored with endocrown restorations featuring 2- and 4-mm pulp chamber extensions had a higher resistance to fracture compared to endocrowns with a 3-mm pulp chamber extension, the fracture resistance mean values were 843.4 N, 943.5 N, and 762.8 N for the three extensions respectively. This difference can be attributed to the different base and endocrown ceramic materials utilized in Hayes et al. [47] and the current study, and primarily the direction of the load to failure, that the study exerted the load on the functional cusp with a 45° angle to the long axis of the tooth, encountering comparable outcomes as Kuijper et al.’s 2020 study [46]. Furthermore, compared to the study by Hayes et al. [47], the current investigation outcomes indicate that the base material used could affect the fracture resistance of ZLS endocrowns. The composite base material improves fracture resistance compared to resin-modified glass ionomer cement (RMGIC) [48,49], regardless of the extent of pulpal extension in the restoration, bringing about more support and less flexion and plastic deformation of the overlying restoration.

When comparing the fracture resistance of teeth with 4 mm extension inlay depths to those with 3 mm depths, it was observed that the former had a lower average fracture resistance. Although this difference was not statistically significant, it is reasonable to attribute the reduced fracture resistance associated with deeper inlay preparations to the increased weakening of the tooth structure. The need to remove more tooth material to accommodate a 4 mm extension inherently compromises the tooth’s structural integrity. Moreover, previous studies have reported that increasing inlay extension leads to an increased endocrown discrepancy [31,50]. As a result, the tooth becomes more susceptible to fractures under stress. Notably, all observed fractures occurred within the tooth structure itself, reinforcing that the depth of the inlay preparation is directly related to the tooth’s ability to withstand force. Preserving as much natural tooth substance as possible is a fundamental principle in restorative dentistry for maintaining the strength and durability of the tooth, and the findings of this comparison support this principle.

Although the pulpal extension depth exhibited a direct correlation to the fracture resistance, utilizing a 1 mm axial ferrule reduced the resistance to fracture of the endocrowns; this could be attributed to the fact that the ferrule design reduced tooth structure and removed a substantial part of the outer circumferential conventional occlusal table of the endocrowns, leading to reduction in the resistance against the compressive loads exerted perpendicular to the occlusal table and parallel to the long axis of the tooth. The ferrule design advantages are well known in the literature to improve the fracture resistance compared with the non-ferrule design, particularly against the obliquely or laterally directed loads; however, the ferrule obviously act in providing more retention and resistance to dislodgement [51], or as a reciprocal part against the laterally exerted stresses as in the case of dynamic occlusal load and lateral excursive occlusal contacts [17,18].

The accuracy of fit of the endocrown restorations is dependent on the design used: the pulpal inlay depth and the presence of the ferrule design, the smallest overall gap encountered by endocrowns without ferrule and with 2 mm pulpal inlay, and the worst gap escorted the endocrowns with 1 mm ferrule design and 4 mm pulpal inlay depth, the lowest gap values were the cervical, axial, and marginal, followed by the internal, and the worst discrepancies were registered at the pulpal floor; detailed values and statistical comparison are shown in Table 2. The discrepancies at the intaglio surfaces (99.6980–122.5640 µm) and the pulpal floor (123–147 µm) were higher than expected. However, all the measured gaps were within the clinically acceptable threshold, according to a common clinical consensus in the past five decades that gap <120 µm is considered acceptable clinically [52,53,54]. The discrepancy of the current study increased with the increase in pulpal depth and design complexity in the endocrown with ferrule groups. The gap values were not directly affected by the prepared designs but by the digital workflow’s limitations and scanning technologies. They faced challenges in capturing and scanning deep inlay walls and floors rather than shallow ones and complex geometrical preparations compared to simple non-ferrule designs. A previous study by Gaintantzopoulou et al. 2016 [55] showed increasing the discrepancy with increasing the pulpal inlay depth of the endocrowns and the complexity of the pulpal floor geometry; these discrepancies were only but a reflection of the scanning inaccuracies and bypassing of complex walls by CNC milling technology [31,55,56]. In the Gaintantzopoulou et al. 2016 study, executed more than 7 years ago, the pulpal floor complex preparations of the canal orifices were bypassed by the digital CNC milling, resulting in even more inaccurate restorations [55].

Nevertheless, a more recent study by Soliman et al. in 2022 [56] reported less than 30 µm mean endocrown gaps for premolar preparations, and the fabricated restorations were able to fit the prepared walls, in particular, the pulpal floor precisely, even with the presence of two small extended studs in the canal orifices. Likewise, another recent study by Gurpinar et al. 2022 [31] reported mean endocrown discrepancies of 2 mm, 3.5 mm, and 5 mm depth to be less than 35 µm. The discrepancy increased with the pulpal inlay depth, depending on the scanning device used [31]. Living in the era of modern dentistry, 120 µm might not be acceptable as an efference for an acceptable, marginal fit in the coming days. The evidence-based conclusions found that no matter the fabrication technique applied, variable dimensions of the marginal gap will always exist. The interface between dentin and luting resin is more susceptible to degradation with a marginal gap of 50–300 microns [57]. Furthermore, irrespective of the risk level, recurrent caries could originate even with 30-micron gaps [58]. Perhaps the time will come to reconsider the upper limit of the clinically acceptable gap threshold, especially with the development and introduction of more advanced scanning devices [31,56] and high-tech production technologies such as 5-axis milling and 3D printing [31,59,60].

The endocrown marginal, axial, and internal fit and gap are governed by many general factors, including predefined cement space value, cement material and professionalism of application, marginal configuration [61], preparation and finish accuracy [62], tooth form and type [63], and inadequate or inappropriate preparation [63,64]. While the fitness of CAD-CAM fabricated endocrowns might be affected by the different software programs and different versions used [65], the type and sophistication of the milling machine (the 5-axis milling over 4- and 3-axis milling machines and the dry milling over wet milling of zirconia) [66,67], and ceramic material type [66]; however, the endocrowns’ accuracy might not be affected by the material when fabricated by 5-axis milling [60]; and thickness of restoration, as thin margins are more prone to chipping [68]. Several ways exist to evaluate the internal gap and marginal fit of crowns and restorations. The current study adopted the silicone replica technique (SRT), while some other studies utilized the triple scan method (TSM) [31], micro-computed tomography (MCT) [55], or digital microscopy [56]. The differences in the accuracy measurement method could explain the different outcomes of these previous studies to the current research, and foremost, the predefined cement space, which in the current study was 60 µm, compared to some studies that might not assigned predefined cement space [31,56].

The fracture resistance values of all the tested groups were high for single restoration, exceeding the upper limit of physiological biting force by far [69]. The conventional endocrown design with a 2–3 mm pulpal inlay might be recommended as a more conservative option. Increasing the endocrown inlay depth might be indicated in situations with high occlusal risk factors, and the ferrule design is beneficial to resist the lateral excursion movement and to withstand the dynamic occlusal cycles [17,18].

The current study has some limitations. Firstly, the applied load was only axial, not considering real-life situations where teeth are subjected to off-axis forces. Secondly, only one type of ceramic material was evaluated. Moreover, the study did not apply thermal aging or cyclic fatigue loading before conducting the fracture resistance test. A post-fatigue fracture resistance test enables the comparison of the residual resistance to a static load on a material subjected to cyclic functional stress [70,71]. To better simulate oral conditions, it would be beneficial to subject the specimens to thermocycling with water, drinks, or artificial saliva. Consequently, further research is needed to examine the failure mechanisms associated with different ceramic materials, teeth, and test conditions under more severe conditions.

## 5. Conclusions

Considering the limitations of the current study, it has been shown that the depth of cavities (intracoronal extensions) significantly impacts the susceptibility of ZLS endocrown restorations to fractures. The findings indicate a clear correlation between greater depth and enhanced fracture resistance. A ferrule design appeared to decrease the resistance of the teeth to fractures when exposed to a vertical load parallel to their long axis. Increasing the depth of the pulpal inlay and incorporating the ferrule design led to decreased accuracy of the restorations’ fit. However, all designs exhibited clinically acceptable marginal, cervical, and axial gaps.

## Figures and Tables

**Table 1:** Materials used in the study.

Trade Name	Scientific Name	Composition	Productive Company
Vita Suprinity	Zirconia-reinforced lithia silicate	SiO_2_ 56–64 wt%, Li_2_O 15–21 wt%, K_2_O 1–4 wt%, P_2_O_5_ 3–8 wt%, Al_2_O_3_ 1–4 wt%, ZrO_2_ 8–12 wt%, CeO_2_ 0–4 wt%, La_2_O_3_ 0–1 wt%, Pigments 0–6 wt%.	VITA Zahnfabrik, Germany
Gradia Core	Dual-cured composite core build-up material	Resin; Bis-GMA, TEGDMA. Filler: Silanated glass, silica Filler loading: (74 wt%) 52 vol% The particle size of 0.04 μm to 23 μm Camphorquinone, Benzoylperoxide	GC America
Clearfill SE	Two-step, self-etch adhesive	Primer: MDP, HEMA, dimethacrylate monomer, water, catalyst Bond: MDP, HEMA, dimethacrylate monomer, microfiller, catalyst	Kuraray America, Houston, TX, USA
Rely X Unicem	Self-adhesive resin cement powder	Glass powder, initiator, silica, substituted pyrimidine, calcium hydroxide, peroxy compound, pigment	3 M ESPE, Seefeld, Germany.
	Self-adhesive resin cement liquid	Methacrylated phosphoric ester, dimethacrylate, acetate, stabilizer, initiator.	

**Table 2:** The mean and standard deviation of the marginal, cervical, axial, pulpal, and internal gaps of endocrowns with different design values are in µm.

Measurements Location		Marginal		Cervical		Axial		Pulpal		Internal	
Endocrown Design		Mean	St. Dev.	Mean	St. Dev.	Mean	St. Dev.	Mean	St. Dev.	Mean	St. Dev.
Butt joint design	2 mm inlay	90.700 ^A^a	11.3925	85.800 ^A^a	8.9790	90.290 ^A^a	4.9328	123.000 ^A^b	14.7573	99.6980 ^A^a	5.64745
	3 mm inlay	105.300 ^B^b	13.2082	88.300 ^A^a	9.3101	94.730 ^B^a	5.4766	138.300 ^B^c	10.2095	107.1110 ^B^b	4.29440
	4 mm inlay	107.100 ^B^b	16.3670	94.500 ^B^a	11.6357	97.360 ^B^a	5.2243	143.400 ^C^c	12.1582	111.7540 ^C^b	5.07847
Ferrule design	2 mm inlay	102.300 ^B^b	13.5158	94.600 ^B^a	10.2220	90.270 ^A^a	2.7105	126.400 ^A^c	11.4426	103.7590 ^B^b	6.82651
	3 mm inlay	116.500 ^C^b	12.7126	109.000 ^C^b	12.8841	95.120 ^B^a	6.7690	138.500 ^B^c	10.2095	115.4080 ^C^b	8.28399
	4 mm inlay	119.500 ^C^b	15.8902	118.100 ^D^b	13.6092	102.590 ^C^a	13.4518	147.000 ^C^b	12.1582	122.5640 ^D^b	10.56441

The superscript uppercase letters represent the statistically significant difference within a column; the small letters represent the statistically significant difference within rows.

**Table 3:** Fracture resistance of endocrowns with and without ferrule at different pulpal inlay depths, the results are given in N.

Endocrown Design	Butt Joint Design		Ferrule Design	
	Mean	St. Dev.	Mean	St. Dev.
**2 mm inlay**	1371.0900 ^A^a	105.48131	1162.1600 ^B^b	375.71287
**3 mm inlay**	1409.6600 ^A^a	49.95278	1246.6100 ^A^b	104.55067
**4 mm inlay**	1396.4833 ^A^a	81.54658	1215.3867 ^A^b	225.40423

The superscript uppercase letters represent the statistically significant difference within a column; the small letters represent the statistically significant difference within rows.

## Data Availability

Data are contained within the article.
